# HiFi long-read amplicon sequencing for full-spectrum variants of human mtDNA

**DOI:** 10.1186/s12864-024-10433-9

**Published:** 2024-05-31

**Authors:** Yan Lin, Jiayin Wang, Ran Xu, Zhe Xu, Yifan Wang, Shirang Pan, Yan Zhang, Qing Tao, Yuying Zhao, Chuanzhu Yan, Zhenhua Cao, Kunqian Ji

**Affiliations:** 1 Research Institute of Neuromuscular and Neurodegenerative Diseases and Department of Neurology, Qilu Hospital, Cheeloo College of Medicine, Shandong University, Jinan, Shandong 250012 China; 2 GrandOmics Biosciences, No.56 Zhichun Road, Haidian District, Beijing, 100098 China; 3 School of Life Sciences, Westlake University, Hangzhou, Zhejiang China; 4 Mitochondrial Medicine Laboratory, Qilu Hospital (Qingdao), Shandong University, Qingdao, Shandong 266035 China; 5 Brain Science Research Institute, Shandong University, Jinan, Shandong 250012 China; 6 Research Institute of Neuromuscular and Neurodegenerative Diseases, Department of Neurology, Qilu Hospital, Shandong University, No. 107 West Wenhua Road, Jinan, Shandong 250012 China

**Keywords:** HiFi sequencing, Mitochondrial disease, mtDNA, SV, SNV

## Abstract

**Background:**

Mitochondrial diseases (MDs) can be caused by single nucleotide variants (SNVs) and structural variants (SVs) in the mitochondrial genome (mtDNA). Presently, identifying deletions in small to medium-sized fragments and accurately detecting low-percentage variants remains challenging due to the limitations of next-generation sequencing (NGS).

**Methods:**

In this study, we integrated targeted long-range polymerase chain reaction (LR-PCR) and PacBio HiFi sequencing to analyze 34 participants, including 28 patients and 6 controls. Of these, 17 samples were subjected to both targeted LR-PCR and NGS to compare the mtDNA variant detection efficacy.

**Results:**

Among the 28 patients tested by long-read sequencing (LRS), 2 patients were found positive for the m.3243 A > G hotspot variant, and 20 patients exhibited single or multiple deletion variants with a proportion exceeding 4%. Comparison between the results of LRS and NGS revealed that both methods exhibited similar efficacy in detecting SNVs exceeding 5%. However, LRS outperformed NGS in detecting SNVs with a ratio below 5%. As for SVs, LRS identified single or multiple deletions in 13 out of 17 cases, whereas NGS only detected single deletions in 8 cases. Furthermore, deletions identified by LRS were validated by Sanger sequencing and quantified in single muscle fibers using real-time PCR. Notably, LRS also effectively and accurately identified secondary mtDNA deletions in idiopathic inflammatory myopathies (IIMs).

**Conclusions:**

LRS outperforms NGS in detecting various types of SNVs and SVs in mtDNA, including those with low frequencies. Our research is a significant advancement in medical comprehension and will provide profound insights into genetics.

**Supplementary Information:**

The online version contains supplementary material available at 10.1186/s12864-024-10433-9.

## Introduction

Mitochondrial diseases (MDs) are a heterogeneous group of disorders involving mutations in mitochondrial DNA (mtDNA) or nuclear DNA (nDNA) [1, 2]. These variants disrupt mitochondrial energy production and have an estimated prevalence of 1 in 5,000 in the general population [3]. To date, over 270 MDs have been classified, with manifestations ranging from mitochondrial encephalopathy and myopathy to multisystemic disorders [4]. The clinical heterogeneity of MDs, coupled with coexisting genetic variability, often complicates their diagnosis and management, leading to poor or late diagnosis [5, 6].

Traditional diagnostic methods lack the efficiency to detect specific variations in mtDNA. The MitoMap (http://www.mitomap.org/MITOMAP) database identifies 94 pathogenic single nucleotide variants (SNVs) and 98 variant types in mtDNA. However, structural variants (SVs), such as deletions and duplications, are less commonly reported, particularly for mid-sized fragments of 300–4000 bp in size. This can be primarily attributed to the limitations of next-generation sequencing (NGS) technologies, which rely on short-read sequencing (SRS) [7].

Long-read sequencing (LRS) technologies, such as single-molecule real-time (SMRT) sequencing and nanopore sequencing, have been commercialized by Pacific Biosciences (PacBio) and Oxford Nanopore Technologies (ONT) [8–11], respectively. LRS has made a transformative impact on genomics by facilitating the sequencing of intricate genomic regions [12]. However, LRS is often limited by high rates of sequencing errors; for instance, SMRT sequencing has reported single-pass error rates of up to 13% [13]. To overcome this problem, novel techniques such as circular consensus sequencing (CCS) and advanced high-fidelity (HiFi) sequencing with improved accuracy have been developed [14–16].

In this study, we combined single-primer long-range polymerase chain reaction (LR-PCR) with PacBio HiFi LRS technology to examine the full-length mtDNA with a high accuracy of 99.9%. Based on our database, GrandmtSVs, we accurately identified mtDNA SNVs and captured complex SVs. The application of improved analytical approaches enhanced the sensitivity of SNV detection, enabling the identification of mutations present at levels below 5%. Furthermore, the pathogenicity of these identified mtDNA SVs in primary mitochondrial diseases were validated through single-muscle fiber sequencing. Noteworthy, we also identified secondary mitochondrial deletions related to idiopathic inflammatory myopathies (IIMs) that could have substantial clinical significance. In conclusion, the integration of LRS technologies can reshape our understanding of MDs, offering unprecedented insights into precision medicine.

## Materials and methods

### Participants and ethical statement

In this study, a total of 34 participants were registered from 2010 to 2023 from Qilu Hospital of Shandong University, China, including 7 cases of IIMs, 6 cases of chronic progressive external ophthalmoplegia (CPEO), 2 cases of mitochondrial encephalomyopathy, lactic acidosis and stroke-like episodes (MELAS), 13 cases of MM, and 6 controls (Table 1). All experimental procedures and methodologies were conducted according to guidelines and regulations. This research was approved by the Ethics Committee of Qilu Hospital, Shandong University, China, and informed consent was obtained from all participants.

**Table 1:** Summary of participants’ information

Participant	Gender	Age	Clinical feature	Sample type
C1	M	13	Control	Muscle tissue
C2	M	35	Control	Muscle tissue
C3	M	7	Control	Muscle tissue
C4	M	14	Control	Muscle tissue
C5	M	37	Control	Muscle tissue
C6	M	54	Control	Muscle tissue
P1	F	13	IIM	Muscle tissue
P2	M	38	IIM	Muscle tissue
P3	F	39	IIM	Muscle tissue
P4	M	40	IIM	Muscle tissue
P5	F	43	IIM	Muscle tissue
P6	F	69	IIM	Muscle tissue
P7	F	35	CPEO	Muscle tissue
P8	F	35	CPEO	Muscle tissue
P9	F	29	CPEO	Muscle tissue
P10	M	61	CPEO	Muscle tissue
P11	M	34	CPEO	Muscle tissue
P12	F	36	CPEO	Muscle tissue
P13	M	47	MM	Muscle tissue
P14	F	46	MM	Muscle tissue
P15	M	32	MELAS	Muscle tissue
P16	F	19	MELAS	Urine
P17	M	39	MM	Muscle tissue
P18	F	14	MM	Muscle tissue
P19	F	25	MM	Muscle tissue
P20	M	27	IIM	Muscle tissue
P21	M	8	MM	Muscle tissue
P22	M	28	MM	Muscle tissue
P23	M	50	MM	Muscle tissue
P24	F	16	MM	Muscle tissue
P25	M	19	MM	Muscle tissue
P26	F	16	MM	Muscle tissue
P27	F	18	MM	Muscle tissue
P28	M	40	MM	Muscle tissue

*Abbreviations* C, control; P, patient; M, male; F, female; IIM, idiopathic inflammatory myopathy; CPEO, chronic progressive external ophthalmoplegia; MELAS, mitochondrial encephalomyopathy, lactic acidosis and stroke-like episodes; MM, mitochondrial myopathy

### Sampling and DNA extraction

DNA was extracted from muscle tissue and urine using a universal DNA extraction kit (D3018-03, Guangzhou Meiji Biotechnology, China), and its quality was assessed using a Qubit 3.0 fluorometer (Q33216, Life Technologies, China) and a NanoDrop 2100 (Agilent, USA).

### Long-range mtDNA amplification

We designed a pair of primers at the conserved D-loop region with reference to the literature published by Wei Zhang et al in 2012 [17]. 5’-CCGCACAAGAGTGCTACTCTCCTC-3’ (chrM:16,426 − 16,448) for the forward primer and 5’-GATATTGATTTCACGGAGGATGGTG-3’ (chrM:16,401 − 16,425) for the reverse primer. The long-range mtDNA amplification was performed by PCR in a 100 µl reaction system. The reaction conditions were as follows: initial denaturation at 94 ℃ for 5 min, followed by denaturation at 98 ℃ for 1 min; annealing at 68 ℃ for 10 min, and a total of 25 cycles, finally 68 ℃ for 20 min. The resulting PCR product was purified using Agencourt AMPure XP magnetic beads (A63882, Beckman Coulter, USA) and subsequently quantified by Qubit (Q33216, Life Invitrogen). Deletions identified by LRS were validated by Sanger sequencing.

### Sanger sequencing

LRS-detected SNVs and SVs were verified by Sanger sequencing. The resulting Sanger sequencing data were carefully analyzed and aligned to the reference genome to ascertain the accuracy of the variants detected by LRS. Discrepancies between the two datasets were flagged for further investigation, and only variants that were confirmed by Sanger sequencing were considered validated. The primer sequences used to validate variations by Sanger sequencing were provide in Table S1.

### NGS and bioinformatics

The amplified mtDNA samples were fragmented into 300–400 bp fragments using an ultrasonic interrupter (KQ218, Kunshan ShuMEI Ultrasonic Instrument, China) and then isolated using Agencourt AMPure XP magnetic beads. The concentration of the isolated DNA was determined using the Qubit dsDNA HS Assay Kit (Q32854, Thermo Fisher Scientific, Waltham, MA), and the fragment size was assessed using the Agilent 2100 system. A DNA library was generated using the Rapid Plus DNA Lib Prep Kit (RK20208, ABclonal, China) and the Dual DNA Adapter 96 Kit for Illumina (RK20287, ABclonal, China) and subjected to high-throughput Illumina NovaSeq technology. The resulting sequencing data were assessed using Illumina Sequence Control Software (SCS) and then subjected to data reading and bioinformatic analysis.

For subsequent processing and analysis, the raw NGS data was first subjected to quality control using *fastp* [18]. *Sentieon* (https://www.sentieon.com/) and *samtools* [19] were employed for comparison, deduplication, and filtering of data with validated quality control. The obtained results were utilized for SNV and SV screening by *Vardict v1.7.0* [20] and *lumpy* [21], respectively. Additionally, *mosdept* [22] was used for statistical analysis of sample depth and coverage maps.

### PacBio sequel sequencing and bioinformatic

The qualified full-length PCR products were used for constructing sequencing libraries using the SMRTbell Express Template Kit 2.0 (PacBio, USA), following the manufacturer’s instructions as follows: ≤ 500 ng cDNA was added to the following components and placed in a thermal cycler with the following program: 37 °C, 30 min; 4 °C, ∞. The repair system was: DNA Prep Buffer 7µL, DNA Damage Repair Mix 2µL, NAD 0.6µL, purified DNA ≤ 47.4µL, rehydration to a total volume of 57µL. After the end of the program, 3µL of End Prep Mix was added, and the mixture was placed in the heat recycler with the following program: 20 °C, 30 min; 65 °C, 30 min; 4 °C, ∞. At the end of the process, the following components were added in a total of 95 µl: Overhang Adapter V3 3µL, Ligation Mix 30µL, Ligation Enhancer 1µL, Ligation Additive 1µL, terminal repair product 60µL. The reaction mixture was placed in the thermal cycler with the following program: 20 °C, 60 min; 4 °C, ∞. The final product was purified using magnetic beads, and its concentration was determined by Qubit dsDNA HS Assay Kit and Agilent 2100.

**Fig. 1:**
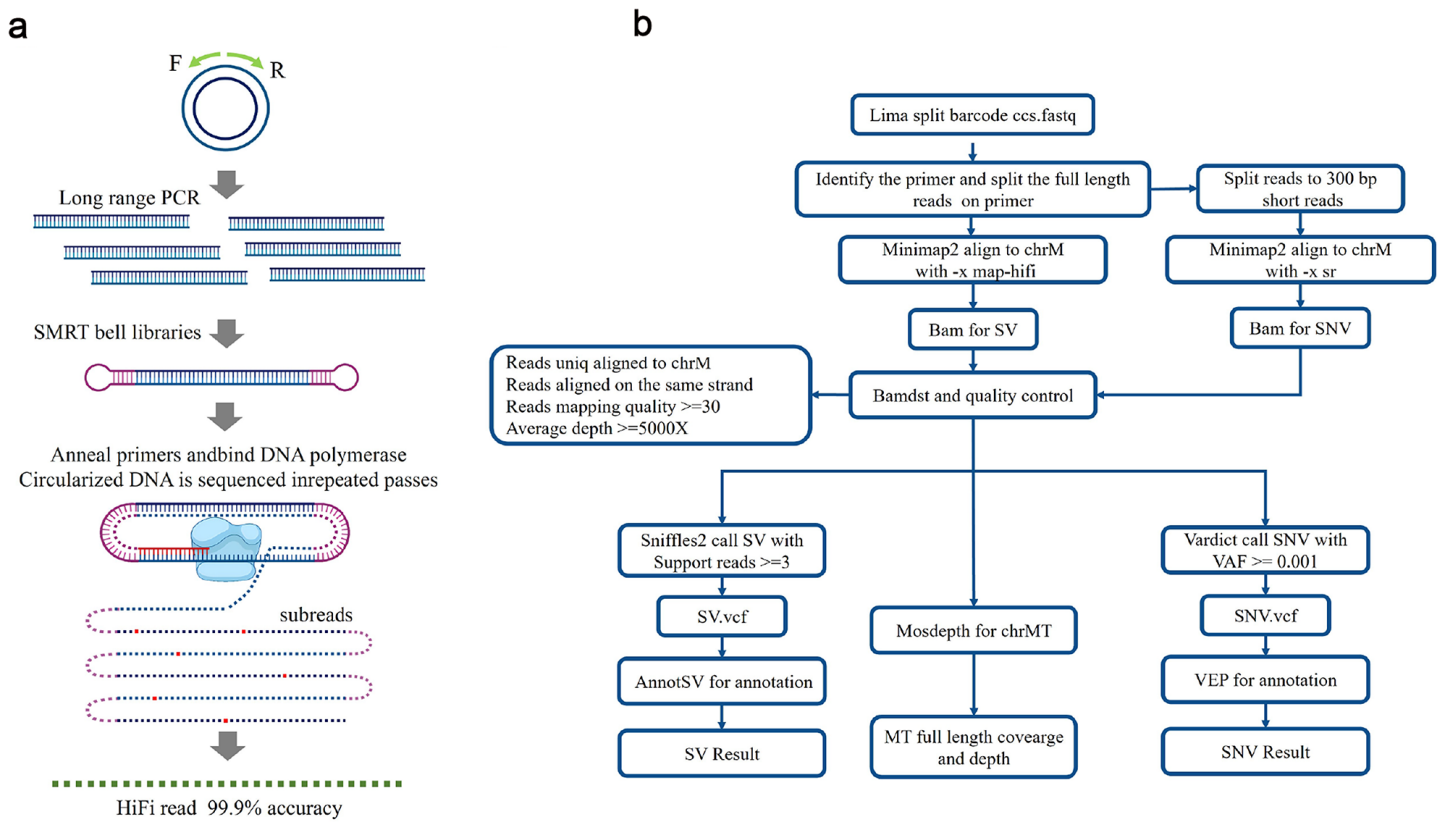
mtDNA PacBio LRS library construction, sequencing, and analysis process. (**a**). Workflow of single-primer long-range PCR and PacBio Sequel II library construction and sequencing. (**b**). Bioinformatics of mtDNA LRS data

Briefly, SMRT bell libraries were generated after DNA damage/terminal repair and hairpin adapter ligation. Following DNA purification using AMPure magnetic beads and concentration measurement by Qubit, the SMRT bell libraries were sequenced by Grand Omics Biosciences (Beijing, China) on a PacBio Sequel II/IIe platform (Fig. 1a). The original sequence data were processed and filtered using the Pacbio SMRT Link® v9.0 standard pipeline (Fig. 1b). The mtDNA sequence data was split according to specific barcodes for each cell. For read alignment, we mapped the Pacbio long reads against the human reference genome (GRCh37/hg19) using *minimap2* [23] and *samtools* [19] to generate the bam file. Meanwhile, we extracted the read-depth information using *mosdepth* [22] to identify SVs; the data without SVs had a minimum minimal depth coverage of 5000x. Then, SVs were called using *Sniffles* [24] and annotated using the *AnnotSV* tool [25]. We then call SNVs and small Indels with *Vardict v1.7.0* [20]. Variants were annotated using the *Ensembl Variant Effect Predictor* [26]. Finally, read count and variant allelic frequency were calculated using bam-read count (https://github.com/genome/bam-readcount) for the supplementation of low-frequency SNVs.

### mtDNA variation analysis

After the screening and annotation of SNV/SV variants, we performed the initial filtering process involving the exclusion of variants with a minor allele frequency < 0.1% and those of low quality to identify potentially deleterious variations. Subsequently, the remaining variants were subjected to further analysis examining their variant status (classified as “confirmed,” “reported,” or “unclear”) using MitoMap. If the variants were either absent or present but not labeled, additional investigation was conducted using other publicly available databases such as MSeqD (https://mseqdr.org/), MtSNPScore (http://ab-openlab.csir.res.in/snpscore/), HmtDB (https://www.hmtdb.uniba.it/), MitoBreak (http://mitobreak.portugene.com/cgi-bin/Mitobreak_home.cgi), and Clinvar (https://www.ncbi.nlm.nih.gov/clinvar/?term=mitochondria+human). This analysis was conducted considering potential deleterious effects, genotype-phenotype associations, scientific literature, and verification through Sanger sequencing.

### Single muscle fiber sequencing

Muscle biopsy specimens of the patient 7 (P7) were obtained following established protocols. After the sample collection, consecutive 20-µm cryosections of the muscle sample were stained using a cytochrome C oxidase (COX) and succinate dehydrogenase (SDH). We meticulously isolated twenty fibers, each exhibiting cytochrome C oxidase positive (COX+) and cytochrome C oxidase negative (COX-) activity, using a tungsten needle. DNA was extracted from two distinct groups: COX + and COX-. To ensure accurate quantification and mitigate potential variations in amplification efficiency between wild-type (WT) and mutant-type (MT) mtDNA, we devised a strategy considering the location of the NADH dehydrogenase 1 (ND1) gene (3307–4262 bp) within the deleted segment and the external location of the NADH dehydrogenase 4 (ND4) gene (10760–12137 bp). The primer sequences designed to amplify corresponding DNA segments are as follows: ND1 forward: 5’-ATGGCCAACCTCCTACTCCT-3’, ND1 reverse: 5’-GCGGTGATGTAGAGGGTGAT-3’; ND4 forward: 5’-CCTGACTCCTACCCCTCACA-3’, ND4 reverse: 5’-GAAGTATGTGCCTGCGTTCA-3’. Each PCR reaction utilized 4 ng of the DNA sample. DNA extracted from normal muscle samples was used as a reference to establish standard curves for the primers, and the analysis involved concentrations ranging from 50 to 0.00032 ng/µl [27, 28]. .

### Pearson’s correlation coefficient

The Pearson correlation coefficient (R) was computed for the ratio in LRS and NGS for each sample, and a scatter plot was generated.

### Statistical analysis

Quantitative data are means ± standard deviations (SDs). The T-test of two independent samples was used for comparison between two groups. Experiments were independently repeated three times. *P* < 0.05 was considered statistically significant, *P < 0.05, **P < 0.01, ***P < 0.001.

## Results

### Cohort characteristics and mtDNA sequencing

**Fig. 2:**
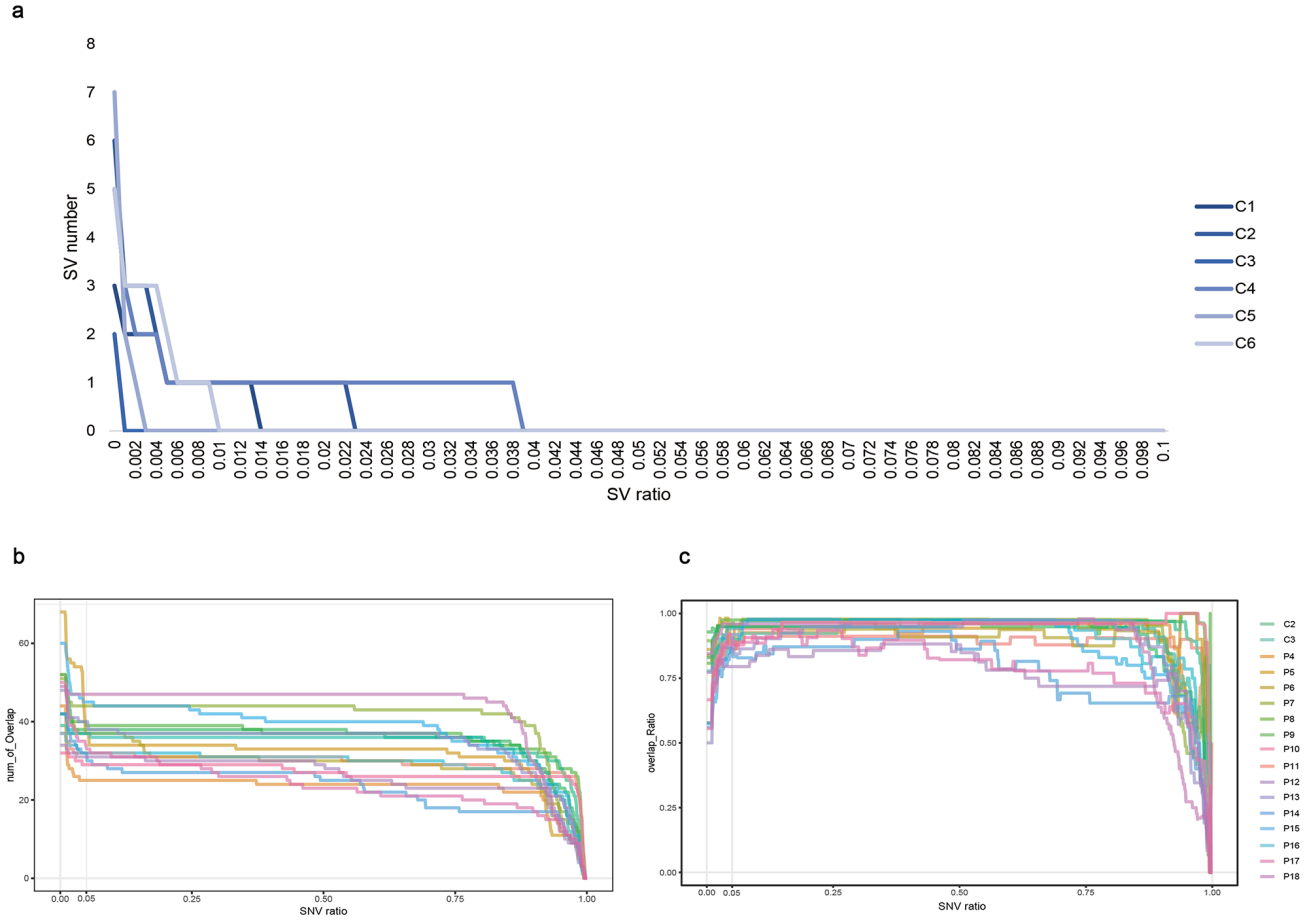
SV and SNV threshold values. (**a**). In 6 controls, the number of SV gradually decreased with the increase of mutation proportion, and the number of SV was 0 for the mutation proportion > 4%. (**b**). Plots showing the number of SNVs shared by LRS and NGS with different ratios. (**c**). The proportion map of SNV shared by LRS and NGS with different ratios

In this study, the cohort of 34 participants included 19 males and 15 females, with an average age of 31.6 years (range: 7–69). Muscle biopsies from 24 patients (P2, P3, P6-P15, P17-P28) revealed mitochondrial abnormalities (Table 1). PacBio LRS was utilized to sequence the muscle tissue or urine of all 34 participants, resulting in a total of 3485.72 Mb raw data and 3240.75 Mb target data. The average coverage for all samples exceeded 5000x. We then performed SNV and SV analyses on 34 samples based on the LRS mitochondrial analysis flow. SNV analysis revealed that two patients (P15 and P16) had mitochondrial hot mutations in MELAS, while no pathogenic SNVs were found in the rest of the participants. Mitochondrial SV analysis does not have the Mitomap’s confirmed pathogenic mutations as SNV, and studies have shown that the common mitochondrial SV is a deletion of 8,470 to 13,447 bp in length associated with CPEO [29]. In addition, studies have reported that mtDNA deletion mutations increase exponentially with age in the normal population [30]. Therefore, we first compared the differences in SVs between 6 controls and 28 patients to determine the threshold proportion of variants in SV that may be associated with MDs. The results showed that there were zero SVs with a ratio of > 4% in 6 controls (Fig. 2a). Deletions with a ratio > 4% were detected in 20 out of 28 patients (Table 2).

**Table 2:** LRS and NGS results of mtDNA

Participants	Gender	Age	Clinical Feature	LRS	NGS	Sanger
C1	M	13	Control	-	×	-
C2	M	35	Control	-	×	-
C3	M	7	Control	-	×	-
C4	M	14	Control	-	×	-
C5	M	37	Control	-	-	-
C6	M	54	Control	-	-	-
P1	F	13	IIM	-	-	-
P2	M	38	IIM	-	-	-
P3	F	39	IIM	chrM:316-166185 del (11.09%)	-	chrM:316-166185 del
P4	M	40	IIM	chrM:2381–16,072 del (56.32%) chrM:3170–16,073 del (20.65%) chrM:5787–16,076 del (7.26%)	chrM:2380–16,070 del (6.75%)	chrM:2380–16,072 del chrM:3168–16,071 del chrM:5786–16,075 del
P5	F	43	IIM	chrM:6751–16,072 del (13.99%) chrM:7821–16,076 del (8.34%)	-	chrM:6750–16,071 del chrM:7280–16,075 del
P6	F	69	IIM	chrM:555-14265 del (5.95%) chrM:3271–16,072 del (13.59%)	-	chrM:543-14265 del chrM:3273–16,073 del
P7	F	35	CPEO	chrM:548–4430 del (18.78%)	-	chrM:542–4436 del
P8	F	35	CPEO	chrM:8569–12,976 del (52.50%) chrM:3271–16,070 del (17.32%)	chrM:8576–12,975 del (60.14%)	chrM:8573–12,979 del chrM:3270–16,068 del
P9	F	29	CPEO	chrM:8470–13,447 del (88.11%)	chrM:8482–13,446 del (53.42%)	chrM:8472–13,448 del
P10	M	61	CPEO	chrM:5787–13,923 del (26.54%) chrM:575–5447 del (5.27%)	chrM:5787–13,922 del (9.74%)	chrM:5788–13,923 del chrM:574–5446 del
P11	M	34	CPEO	chrM:5787–13,923 del (47.68%)	chrM:5781–13,922 del (3.82%)	chrM:5787–13,923 del
P12	F	36	CPEO	chrM:3264–12,299 del (16.02%) chrM:807-14901 del (5.51%)	chrM:3263–12,298 del (6.70%) chrM:807-14901 del (7.00%)	chrM:3264–12,299 del chrM:804-14900 del
P13	M	47	MM	chrM:5788–13,923 del (6.78%)	chrM:5787–13,922 del (3.26%)	chrM:5788–13,923 del
P14	F	46	MM	chrM:5788–13,923 del (42.47%)	chrMT:5787–13,922 del (13.33%)	chrM:5788–13,922 del
P15	M	32	MELAS	MT-TL1 m.3243 A > G (81.33%) chrM:497-14330 del (32.50%)	MT-TL1 m.3243 A > G (98.05%)	chrM:495-14334 del MT-TL1 m.3243 A > G (99.99%)
P16	F	19	MELAS	MT-TL1 m.3243 A > G (35.65%)	×	MT-TL1 m.3243 A > G (32.1%)
P17	M	39	MM	chrM:315-16185 del (14.96%)	×	chrM: 315-16185 del
P18	F	14	MM	chrM:316-16185 del (18.25%)	×	chrM:316-16185 del
P19	F	25	MM	-	×	-
P20	M	27	IIM	chrM:3264–12,299 del (12.60%)	×	chrM:3264–12,299 del
P21	M	8	MM	-	×	-
P22	M	28	MM	-	×	-
P23	M	50	MM	-	×	-
P24	F	16	MM	chrM:3264–16,068 del (18.25%) chrM:2167–13,923 del (8.23%)	×	chrM:3264–16,068 del chrM:2167–13,923 del
P25	M	19	MM	chrM:3264–14,413 del (18.42%)	×	NA
P26	F	16	MM	-	×	-
P27	F	18	MM	chrM:351-16071 del (24.03% ) chrM:3273–15,882 del (12.81%) chrM:1693–13,923 del (9.13%) chrM:1773–14,262 del (7.80%)	×	NA
P28	M	40	MM	chrM:3264–13,923 del (8.69%) chrM:3264–16,072 del (27.77%)	×	NA

*Abbreviations* C, control; P, patient; M, male; F, female; CPEO, chronic progressive external ophthalmoplegia; MELAS, mitochondrial encephalomyopathy, lactic acidosis and stroke-like episodes; MM, mitochondrial myopathy; IIM, idiopathic inflammatory myopathy. Dash del (-) denotes SV with a ratio > 4% was not detected. × denotes NGS sequencing was not performed. NA means the sample size was insufficient to support validation

### Comparison of SNV results between NGS and LRS

NGS is a popular method for the identification of mtDNA SNVs. Consequently, we aimed to assess and compare the efficacy of LRS and NGS in detecting SNVs. We conducted a comprehensive analysis of SNV results from 17 participants (C5-P15) who underwent both SRS and LRS. Initially, we evaluated the concordance between the two data sets. *Vardict v1.7.0* was employed for SNV calling after quality control of the NGS and LRS data. The findings indicated that the proportion of SNVs with a ratio < 5% was higher in all LRS samples compared to NGS (Fig. S1a). Meanwhile, SNVs with a ratio > 5% were consistent between LRS and SRS (Fig. 2b). Additionally, the average Pearson correlation R-value of about 0.94607 (ranging from 0.7578 to 0.9988, Fig. S1b) indicated no discernible difference between LRS and SRS for SNVs with a ratio > 5%. Additionally, we compared other heterogeneity loci and found no significant differences (Table S2). For example, in the patient 15 (P15), both LRS and NGS detected m.3243 A > G hotspot variation, with a ratio of 81.33% and 98.05%, respectively, while LRS also detected chrM: 497-14330 deletion with a ratio of 32.50%, which also explained the reason why LRS detected a lower ratio at m.3243 A > G than NGS, highlighting the superiority of LRS in detecting mtDNA multiple variation. The combined analysis consistently demonstrated no discrepancy in the detection of SNVs. However, LRS outperformed NGS in identifying low-frequency SNVs with a ratio below 5%.

### Comparison of SV results between NGS and LRS

#### Multiple deletions

**Fig. 3:**
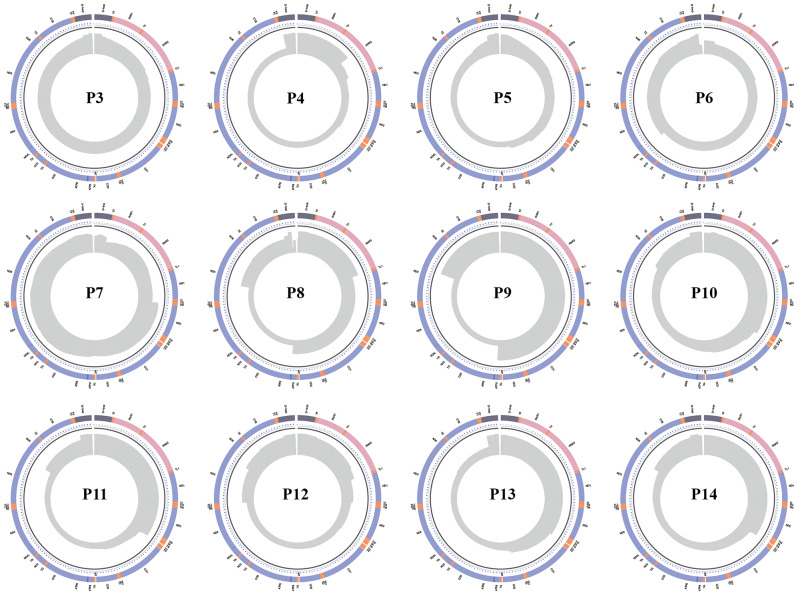
Statistical circles of patients 3–14 (P3-P14) read depth. The outer circle shows the mitochondrial gene distribution map; purple, pink, orange, and dark gray mark mitochondrial genes, rRNA, tRNA, and the D-loop region, respectively. The middle represents the mitochondrial 16,569 bp coordinate, and the gray area in the inner circle denotes the distribution of the reads, which was used to observe SV

In the patient 8 (P8), mtDNA deletions were detected at positions chrM: 8568–12,976 and chrM: 3270–16,070 by LRS, with variation ratios of 52.50% and 17.32%, respectively. These deletions were subsequently confirmed by Sanger sequencing. In contrast, NGS only identified the chrM: 8568–12,976 deletion with a variation ratio of 60.14%. Notably, a statistical analysis of LRS read depth effectively distinguished between the two mtDNA deletions (Fig. 3). Furthermore, in patients 4 and 10 (P4 and P10), LRS detected multiple mtDNA deletions, whereas NGS only identified a single deletion. This highlights the superior accuracy of LRS in detecting mtDNA structural variations, facilitating investigation into the underlying mechanisms of MDs.

#### Full-frequency SV

In patients P9, P11, P12, P13, and P14, both LRS and NGS identified the same deletion variations (chrM: 8470–13,447, chrM: 5787–13,923, chrM: 575–5447, chrM: 3264–12,299, and chrM: 807-14901). However, LRS detected a higher proportion of variations than NGS. Conversely, in patients P3, P5, P6, and P7, LRS could detect mtDNA deletions while NGS failed to do so. The presence of these deletions was validated by Sanger sequencing (Table 2; Fig. 3). Additionally, upon reanalyzing the SV data obtained from NGS, we found that LRS-identified deletions exhibited a very low proportion of variants in NGS results. These findings indicated that LRS offers a comparative advantage over NGS for the identification of SVs with a lower proportion. Concisely, LRS is more suitable for detecting full-frequency SV.

### Quantitative analysis of SV in single muscle fibers using real-time PCR

**Fig. 4:**
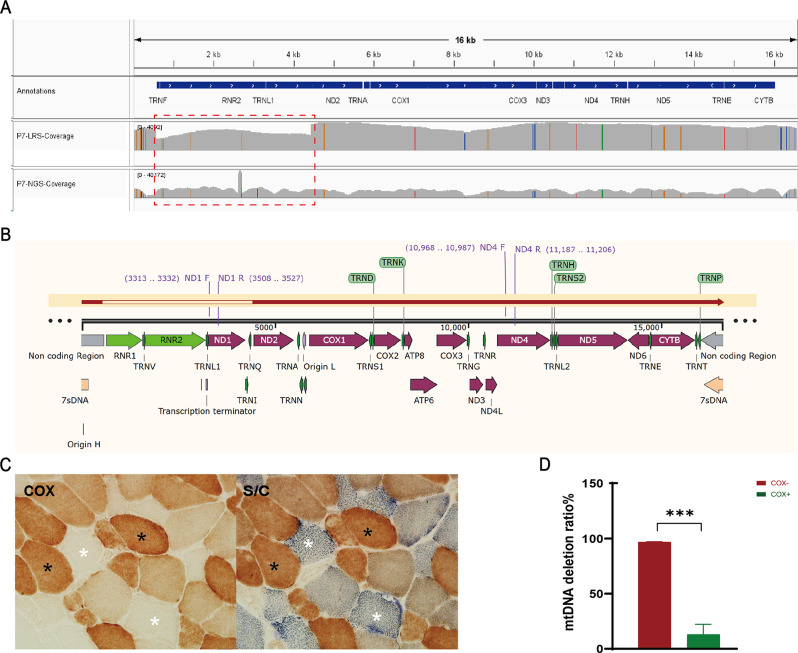
Quantification of the level of a 3881 bp (chrM: 548-4439del) mtDNA deletion at the single cell level. (**A**). Integrative Genomics Viewer (IGV) view of depth data for LRS and NGS at P7. The red box is a deletion indicated by the LRS depth data (**B**). Comparison of deleted mtDNA and complete mtDNA; the exact location of primers is marked in the figure. (**C**). Serial sections of the patient’s muscle sample with COX and S/C staining. COX-negative (COX-) and COX-positive (COX+) fibers are marked. (**D**). The ratio of mtDNA deletion in two groups: COX-, and COX+

To verify whether the proportion of SV variation detected by LRS was pathogenic, we performed a quantitative analysis of SV in individual muscle fibers by real-time PCR on muscle tissue samples from the patient 7 (P7; chrM: 548–4430 del). The standard curves of primer ND1 and ND4 as well as the computational formula for mtDNA deletion ratio were provided in Fig. S2 in detail. As shown in Fig. 4, the mtDNA deletion rate in the combination of COX- group was significantly higher compared to the COX + group, with values of 96.6% and 11.9%, respectively, which suggested the pathogenicity of this SV. When comparing the depth statistics of NGS and LRS within the same IGV window and under identical conditions, it was observed that the deep coverage data from LRS clearly indicated the presence of mitochondrial genome deletions in P7 patients, characterized by distinct breakpoints. In contrast, the deep coverage data from NGS did not provide any indication of such deletions. Specifically, LRS detected a proportion of 18.78% of mtDNA deletions, whereas NGS data did not reveal any deletions (Fig. 4A). These results suggested the sensitivity of LRS to identify SVs, however, the pathogenicity of these identified SVs still needs to be further verified.

### Application of LRS in inflammatory myopathy

**Fig. 5:**
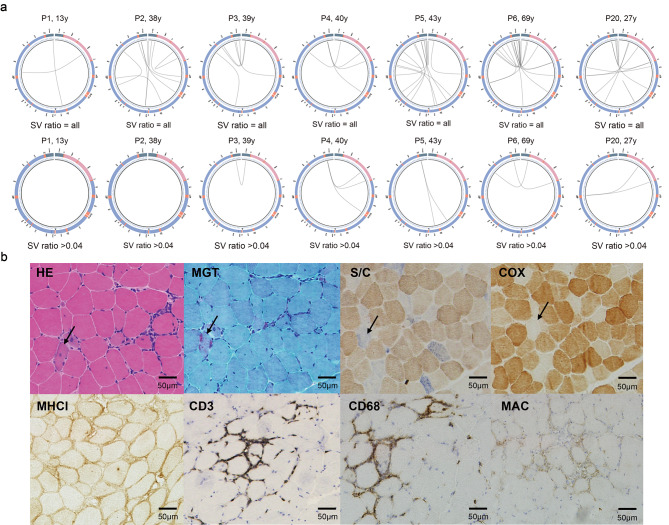
SV circle diagram of myositis patient and muscle histological and histochemical pathological images of P20. (**A**). P1-P6, and P20 all were detected with SV and SV with a ratio > 4% circle plots. (**B**). Muscle histology and histochemistry suggested mitochondrion dysfunctions in P20. In the first-line pictures, HE, MGT, COX, and SDH/COX double staining showed the features of mitochondrial dysfunctions. In the second line, the infiltrates of CD3^+^ and CD68^+^ cells, along with the expressions of MHC-1 and MAC, were consistent with pathological changes in inflammatory myopathy. HE: hematoxylin and eosin; MGT: modified Gomori trichrome; COX: cytochrome C oxidase; SDH: succinate dehydrogenase; S/C: SDH/COX double histochemistry; MHC-I: anti-major histocompatibility complex class I; MxA: myxovirus resistant protein A

To test the accuracy and sensitivity of LRS in other neuromuscular diseases that are usually accompanied by secondary mitochondrial damage, we enrolled 7 IIMs patients (P1-P6, P20), aged 13–69 years (Table 1). 4 out of 7 patients exhibited mitochondrial dysfunctions in muscle biopsy (Fig. 5 and Fig. S3). NGS detected mtDNA deletion in only one patient with a low ratio. In contrast, LRS detected multiple deletions with a higher ratio in P3-P6 and P20 (Fig. 5A). All these mtDNA deletions were confirmed by Sanger sequencing. These results indicated the superiority of LRS over NGS in detecting multiple mtDNA deletions not only in primary MDs but also in secondary mitochondrial dysfunctions.

## Discussion

MtDNA sequencing plays a critical role in mitochondrial genetics, evolution, and disease diagnosis. Conventional methods for detecting mtDNA include Sanger sequencing (used as a benchmark for identifying single gene point variants), Southern blot (for detecting mtDNA deletions), and real-time PCR (for determining DNA copy number). Nevertheless, these methods have limitations regarding throughput, sensitivity, and speed.

NGS technology has gained popularity for identifying clinical genetics and MDs due to its high throughput and rapid detection capabilities. However, the mitochondrial genome, which contains multiple copies of mtDNA within a cell, poses challenges for NGS in determining whether aligned reads originate from the same mtDNA molecule. Accurate detection of small and medium-sized deletions, as well as low proportions of SVs, remains a limitation in NGS analysis of mitochondrial genomes. Another constraint in analyzing mitochondrial genomes using NGS is the presence of nuclear mitochondrial sequences (NUMTs) [31]. NUMTs refer to fragments of mtDNA incorporated into the nuclear genome sequence during eukaryotic evolution. The length of most human NUMTs exceeds that of reads generated by NGS, making it challenging to distinguish between mtDNA and NUMTs through sequence alignment. This ambiguity can lead to false negative or false positive results and errors in quantifying heterogeneity [32, 33].

With the advent of ONT and PacBio LRS technologies, it is now possible to obtain reads spanning the entire 16,569 bp length of mtDNA. This comprehensive coverage enables the detection of all SNVs, insertions, deletions (Indels), and structural SVs present in mtDNA. In a recent study, ONT MinION sequencing was used for LRS on nine patients with mitochondrial genome deletions and three controls without MD phenotypes. The findings demonstrated that ONT MinION LRS improved the analysis of large fragment deletions and complex rearrangements in mtDNA compared to SRS using NGS methods. However, it should be noted that the accuracy of ONT MinION LRS in identifying single base variations is limited, especially in homopolymeric stretches [34, 35].

Similarly, the analysis of the mitochondrial genome of the silky shark *Carcharhinus* falciformis, equine, and *Oryctes rhinoceros* using the ONT LRS method also indicated errors in single base calling and homopolymer runs. In comparison to ONT, PacBio enhances the precision of Single molecule real-time (SMRT) sequencing and produces long HiFi readings with optimized CCS, resulting in a higher accuracy rate of 99.9%. This makes PacBio more suitable for identifying mitochondrial SNVs, Indels, and SVs [15]. A study of the Tibetan Mastiff mitochondrial PacBio LRS showed that the average accuracy of HiFi reads could reach 99.6% (phred quality score 24, Q24). If two rounds of CCS were performed, the accuracy of HiFi reads could even reach 99.999% (Q50) [36]. PacBio LRS has identified species-specific structural unit sequences not found in previous animal mitochondrial assembly studies, such as *Echinococcus granulosus* [37], and *Potamopyrgus* [38], providing a reference for PacBio in human mitochondrial genome sequencing research.

Reported methods for mtDNA enrichment include long-range PCR [17], exonuclease and rolling circle amplification [39], LostArc [40], and nanopore Cas9-targeted sequencing (nCATS) [9, 41]. In this study, a pair of primers long-range PCR was used to obtain the full length of mtDNA. In contrast to utilizing multiple primers in NGS, the use of a single pair of primers can help alleviate variations in PCR efficiency across different amplicons, thereby reducing the likelihood of rare or novel mutations occurring at primer binding sites. Furthermore, the design of primers targeting the conserved D-loop region aims to circumvent documented point mutations and hotspot deletion mutations within the coding region of genes, facilitating comprehensive and consistent detection of mutations within the mitochondrial genome. While infrequent, it is important to acknowledge the potential limitations associated with designing primers at the D-loop region for LR-PCR. It is acknowledged that the identification of deletions or variations within this region may pose difficulties, potentially compromising the accuracy of variant validation at the primer region.

To carry out a comprehensive genetic interpretation for patients with clinical and pathological indications of MDs, we employed full-length HiFi sequencing. This advanced sequencing method revealed the presence of one or multiple substantial fragment deletions, exceeding 5% in magnitude, in both the cohort of six patients diagnosed with CPEO and the group of ten patients with mitochondrial myopathy (MM). Interestingly, the investigation of certain deletions, particularly those with proportions less than 10%, proved to be challenging using NGS in specific patients from both groups. In sharp contrast, our control groups did not manifest any mtDNA deletions.

Additional validation at the single-fiber level was conducted to confirm the presence of mtDNA deletions identified through LRS. Notably, COX- fibers displayed a significantly higher prevalence of deletions compared to COX + fibers. This finding highlights the potential pathogenic nature of these recently discovered mtDNA deletions, implying their potential contribution to the observed clinical phenotypes in affected patients.

LRS also have diagnostic value in secondary mitochondrial damage-related diseases, such as IIMs. Previous studies had documented that IIMs can be associated with mitochondrial damage. Additionally, literatures highlighted a positive correlation between vascular injury, infiltration of inflammatory cells, and the presence of COX- muscle fibers [42–44]. In this study, we used sensitive LRS and detected nine types of mtDNA deletions from five IIMs patients. Muscle ischemia, aging, and other factors related to IIMs can induce mtDNA damage, eventually, causing cellular respiratory dysfunction, atrophy, and fiber degeneration [45]. Therefore, it is vital to evaluate and assess the impact of mtDNA damage, particularly, in the diagnosis and management of IIMs. The full-length HiFi sequencing, with its increased precision, can accurately evaluate mtDNA damage in such patients, enabling a more comprehensive understanding of the underlying molecular mechanisms.

In summary, the application of single-primer LR-PCR combined with PacBio HiFi LRS has been employed for the initial detection of human mtGenes. Our findings indicate that this approach can effectively identify both SNVs and SVs of full frequency and multiple types across the entire mitochondrial genome. Although the precise association between numerous SVs and MDs remains undetermined, the increasing utilization of LRS in mitochondrial analysis, coupled with the accumulation of case studies, will contribute to a clearer and more definitive understanding of the pathogenic mechanisms underlying mitochondrial genomic variants.

### Electronic supplementary material

Below is the link to the electronic supplementary material.

Supplementary Material 1

Supplementary Material 2

Supplementary Material 3

Supplementary Material 4

Supplementary Material 5

Supplementary Material 6

## Data Availability

All sequencing reads will be accessible with the following link: https://www.ncbi.nlm.nih.gov/sra/PRJNA1082905. The accession number is PRJNA1082905.
