# Correction: HiFi long-read amplicon sequencing for full-spectrum variants of human mtDNA

**DOI:** 10.1186/s12864-024-10660-0

**Published:** 2024-07-30

**Authors:** Yan Lin, Jiayin Wang, Ran Xu, Zhe Xu, Yifan Wang, Shirang Pan, Yan Zhang, Qing Tao, Yuying Zhao, Chuanzhu Yan, Zhenhua Cao, Kunqian Ji

**Affiliations:** 1 Research Institute of Neuromuscular and Neurodegenerative Diseases, Department of Neurology, Cheeloo College of Medicine, Qilu Hospital, Shandong University, Jinan, Shandong 250012 China; 2 GrandOmics Biosciences, No.56 Zhichun Road, Haidian District, Beijing, 100098 China; 3 School of Life Sciences, Westlake University, Hangzhou, Zhejiang China; 4 Mitochondrial Medicine Laboratory, Qilu Hospital (Qingdao), Shandong University, Qingdao, Shandong 266035 China; 5 Brain Science Research Institute, Shandong University, Jinan, Shandong 250012 China; 6 Research Institute of Neuromuscular and Neurodegenerative Diseases, Department of Neurology, Qilu Hospital, Shandong University, No. 107 West Wenhua Road, Jinan, Shandong 250012 China

**Correction: BMC Genomics 25**,** 538 (2024)**

10.1186/s12864-024-10433-9

In this article, an older version of Figure 5 was submitted for publication. The correct and incorrect version of Fig. 5 is given below. The figure caption remains unchanged.

Correct

**Fig. 5:**
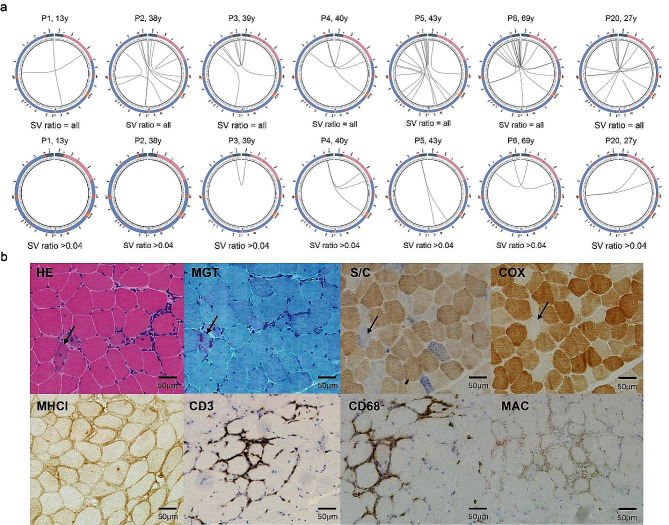
SV circle diagram of myositis patient and muscle histological and histochemical pathological images of P20. (**A**). P1-P6, and P20 all were detected with SV and SV with a ratio > 4% circle plots. (**B**). Muscle histology and histochemistry suggested mitochondrion dysfunctions in P20. In the first-line pictures, HE, MGT, COX, and SDH/COX double staining showed the features of mitochondrial dysfunctions. In the second line, the infiltrates of CD3^+^ and CD68^+^ cells, along with the expressions of MHC-1 and MAC, were consistent with pathological changes in inflammatory myopathy. HE: hematoxylin and eosin; MGT: modified Gomori trichrome; COX: cytochrome C oxidase; SDH: succinate dehydrogenase; S/C: SDH/COX double histochemistry; MHC-I: anti-major histocompatibility complex class I; MxA: myxovirus resistant protein A

Incorrect

**Figure:**
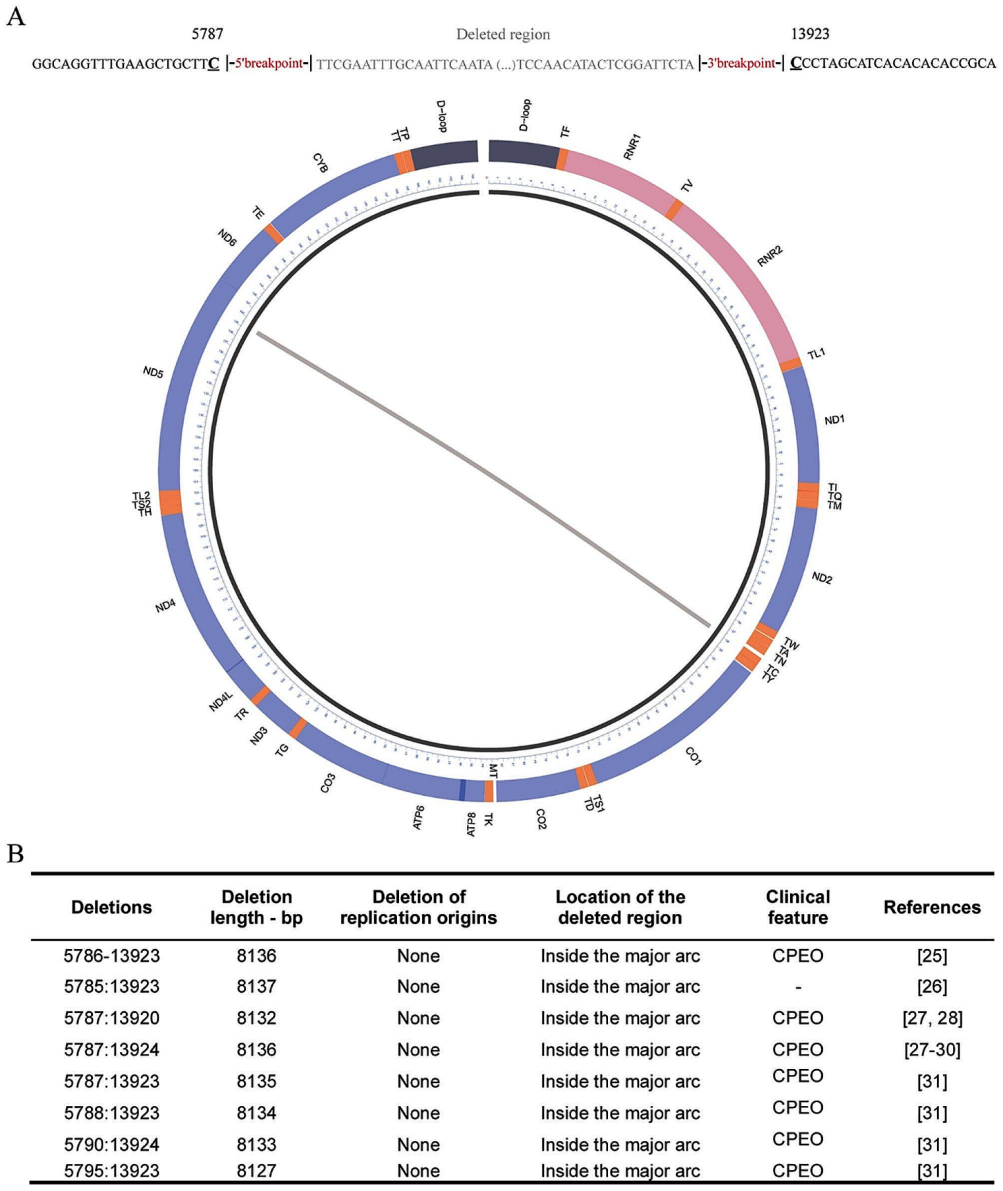

The original article has been corrected.

The authors would like to apologize for any inconvenience caused to the readers.

