# Supplementary figures and images for: HiFi long-read amplicon sequencing for full-spectrum variants of human mtDNA
Source: BMC Genomics. 2024 May 31;25:538. doi: 10.1186/s12864-024-10433-9 (PMC11141058; doi:10.1186/s12864-024-10433-9)

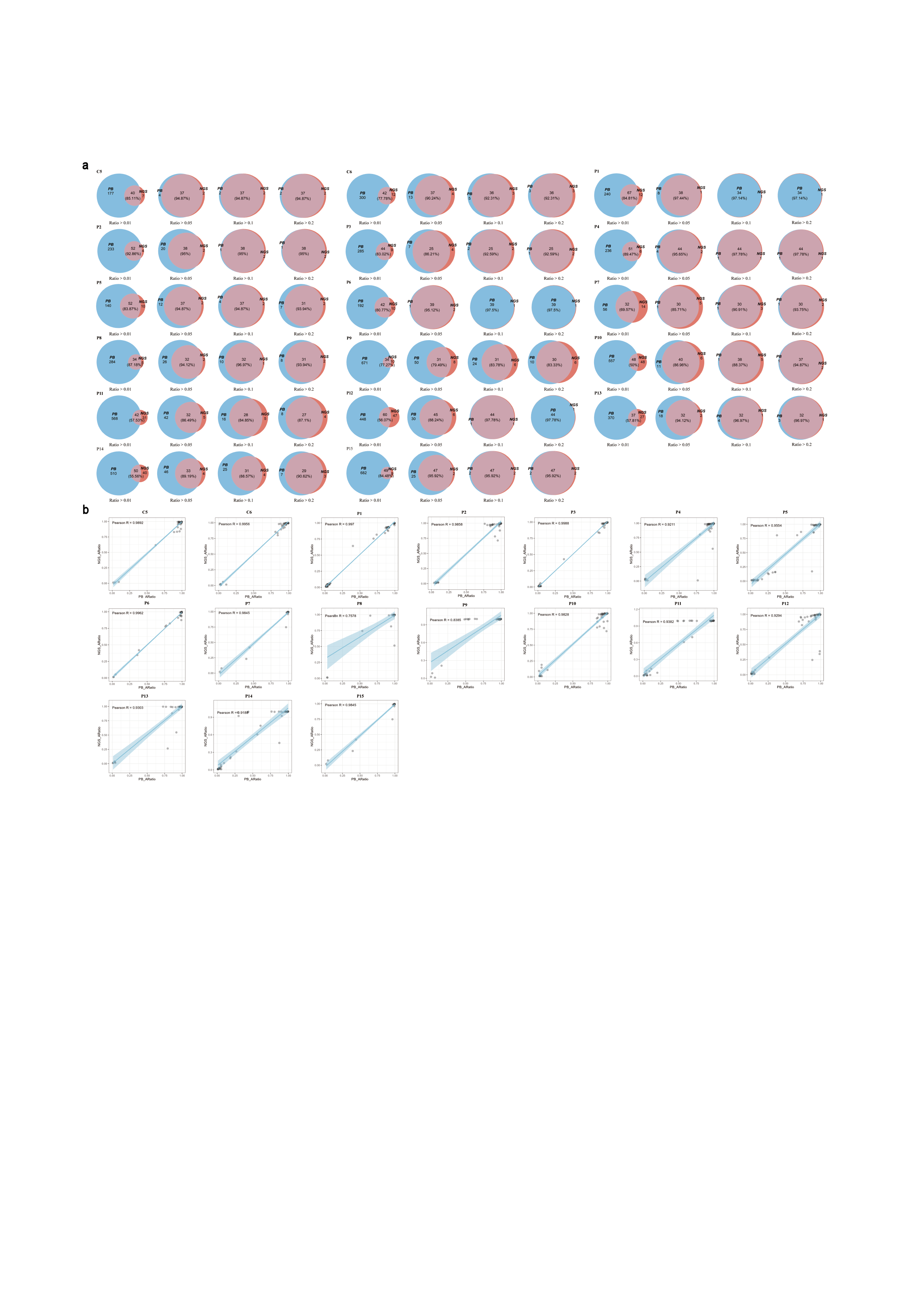

Supplement: Supplementary file 1 — Supplementary Material 1 [file 12864_2024_10433_MOESM1_ESM.tif]

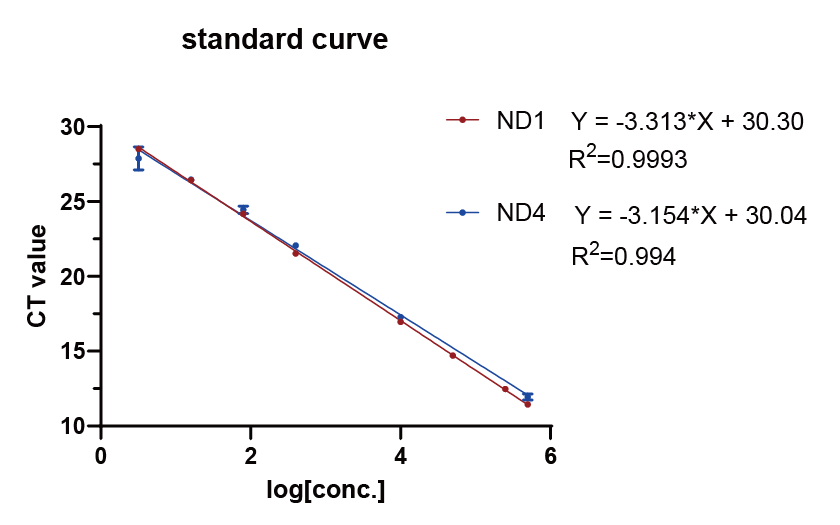

Supplement: Supplementary file 2 — Supplementary Material 2 [file 12864_2024_10433_MOESM2_ESM.tif]

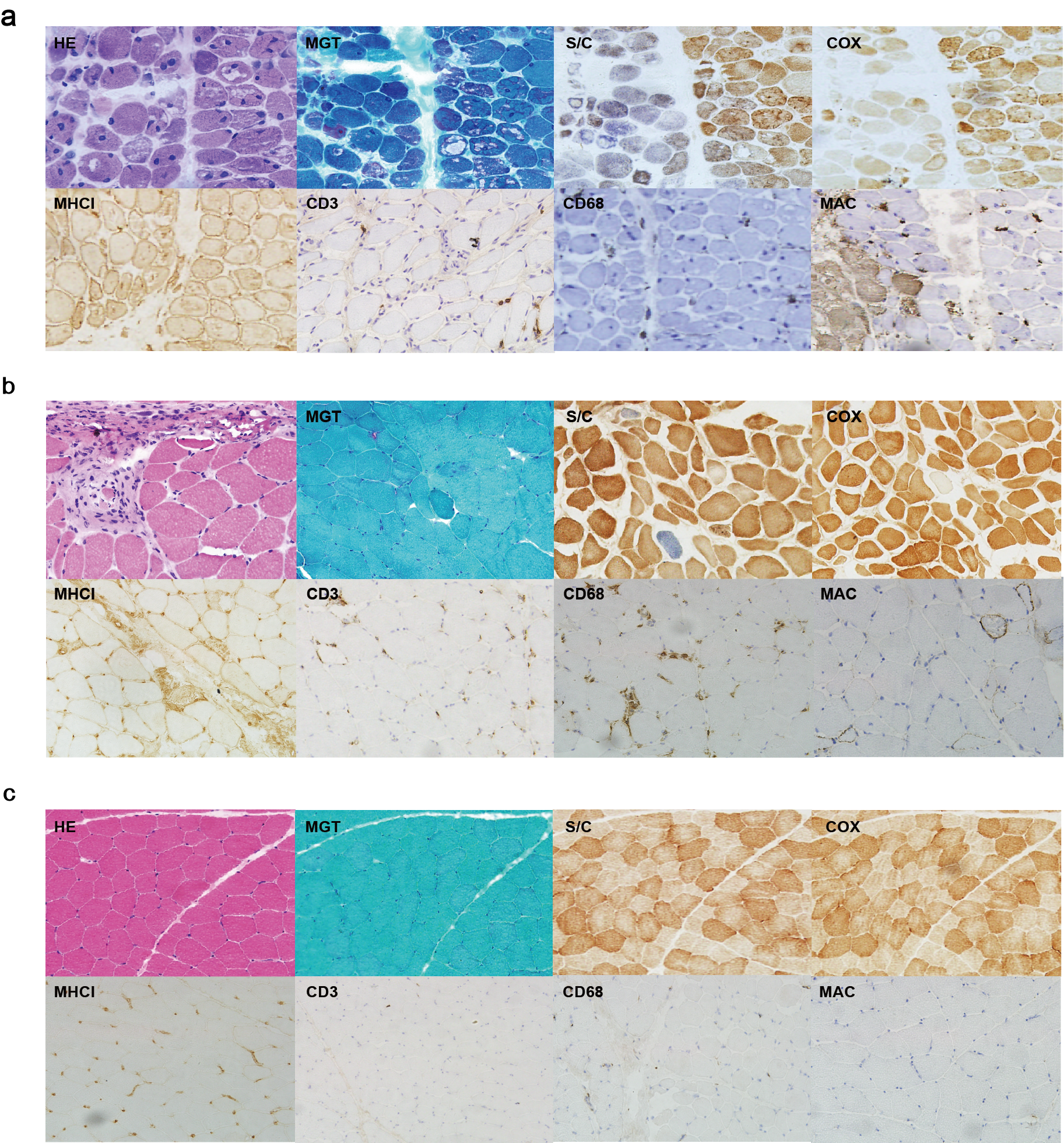

Supplement: Supplementary file 3 — Supplementary Material 3 [file 12864_2024_10433_MOESM3_ESM.tif]
