# Supplementary material for: HiFi long-read amplicon sequencing for full-spectrum variants of human mtDNA
Source: BMC Genomics. 2024 May 31;25:538. doi: 10.1186/s12864-024-10433-9 (PMC11141058; doi:10.1186/s12864-024-10433-9)
Supplement: Supplementary file 4 — Supplementary Material 4 [file 12864_2024_10433_MOESM4_ESM.docx]

**Supplementary figure legends**

**Fig. S1 Comparison of SNV results between NGS and LRS with Venn plots and correlation coefficient plots. a.** Venn diagram of the number of shared or unique SNVs with the ratio >1%, >5%, >10%, and >20% of NGS and LRS detected. **b.** Pearson correlation coefficient plots for NGS and LRS.

**Fig. S2 The standard curves of ND1 and ND4 as well as the computational formula for mtDNA deletion ratio.** The resulting threshold cycle (CT) values for the standard curves of ND1 and ND4 ranged from 11.4 to 28.6 and 11.5 to 27.8, respectively. A linear relationship between the CT values and the logarithm of the concentration indicated a direct association with the initial copy numbers of ND1 and ND4. The mtDNA quantities in individual samples were determined based on these findings and found to be within the linear range, indicating the accurate measurement of ND1 and ND4 levels. The similar amplification efficiency of the ND1 and ND4 fragments enabled the use of CT values in determining their relative copy number. The mtDNA deletion ratio was calculated using the equation: R = 1 - (ND1/ND4), where R represents the ratio.

**Fig. S3 Muscle biopsy of IIM patients with mitochondrial dysfunctions and normal control. a. and b.** Muscle histology and histochemistry suggested mitochondrion dysfunctions in some IIM patients. **c.** Muscle biopsy of normal control.
