# Supplementary material for: HiFi long-read amplicon sequencing for full-spectrum variants of human mtDNA
Source: BMC Genomics. 2024 May 31;25:538. doi: 10.1186/s12864-024-10433-9 (PMC11141058; doi:10.1186/s12864-024-10433-9)
Supplement: Supplementary file 5 — Supplementary Material 5 [file 12864_2024_10433_MOESM5_ESM.docx]

**Supplementary Table 1. The primer sequences used to validate deletions by Sanger sequencing.**

| **Participant** | **Clinical Feature** | **LRS** |  |
| --- | --- | --- | --- |
| C1 | Control | - | - |
| C2 | Control | - | - |
| C3 | Control | - | - |
| C4 | Control | - | - |
| C5 | Control | - | - |
| C6 | Control | - | - |
| P1 | IIM | - | - |
| P2 | IIM | - | - |
| P3 | IIM | chrM:316-166185 del (11.09%) | F:GTATGCACGCGATAGCATTG  R:GCACTCTTGTGCGGGATATT |
| P4 | IIM | chrM:2381-16072 del (56.32%) | F:TGGTGATAGCTGGTTGTCCA  R:GCACTCTTGTGCGGGATATT |
|  |  | chrM:3170-16073 del (20.65%) | F:GGGATAACAGCGCAATCCTA  R:GCACTCTTGTGCGGGATATT |
|  |  | chrM:5787-16076 del (7.26%) | F:ACACTCATCGCCCTTACCAC  R:GCACTCTTGTGCGGGATATT |
| P5 | IIM | chrM:6751-16072 del (13.99%) | F:CTTAGGGGCCATCAATTTCA  R:GTGGCTTTGGAGTTGCAGTT |
|  |  | chrM:7821-16076 del (8.34%) | F:ACATGCAGCGCAAGTAGGTC  R:GTGGCTTTGGAGTTGCAGTT |
| P6 | IIM | chrM:555-14265 del (5.95%) | F:CTGGCCACAGCACTTAAACA R:TCCGTGCGAGAATAATGATG |
|  |  | chrM:3271-16072 del (13.59%) | F:GGGATAACAGCGCAATCCTA  R:GCACTCTTGTGCGGGATATT |
| P7 | CPEO | chrM:548-4430 del (18.78%) | F:GTATGCACGCGATAGCATTG  R:TCAGAAGTGAAAGGGGGCTA |
| P8 | CPEO | chrM:8569-12976 del (52.50%) | F:CATGCCCATCGTCCTAGAAT  R:TTGGTTGATGCCGATTGTAA |
|  |  | chrM:3271-16070 del (17.32%) | F:GGGATAACAGCGCAATCCTA  R:GCACTCTTGTGCGGGATATT |
| P9 | CPEO | chrM: 8470-13447 del (88.11%) | F:TGGAGCAAACCACAGTTTCA  R:GCGAGGGCTGTGAGTTTTAG |
| P10 | CPEO | chrM: 5787-13923 del (26.54%) | F:GGCCATTATCGAAGAATTCACR:TTTAGGGGGAATGATGGTTG |
|  |  | chrM:575-5447 del (5.27%) | F:TTTGATTCCTGCCTCATCCT R:GGGTGAGGTAAAATGGCTGA |
| P11 | CPEO | chrM: 5787-13923 del (47.68%) | F:ACACTCATCGCCCTTACCAC  R:CGGGGGAATAGGTTATGTGA |
| P12 | CPEO | chrM: 3264-12299 del (16.02%) | F:GGGATAACAGCGCAATCCTA  R:TGGCTCAGTGTCAGTTCGAG |
|  |  | chrM:807-14901 del (5.51%) | F:CCCTAACACCAGCCTAACCA  R:AATGTATGGGATGGCGGATA |
| P13 | MM | chrM:5788-13923 del (6.78%) | F:AATTCCATCCACCCTCCTCT  R:AGGTAGGATTGGTGCTGTGG |
| P14 | MM | chrM: 5788-13923 del (42.47%) | F:AATTCCATCCACCCTCCTCT  R:AGGTAGGATTGGTGCTGTGG |
| P15 | MELAS | MT-TL1 m.3243A>G (81.33%)  chrM: 497-14330 del (32.50%) | F1:AATCCAGGTCGGTTTCTATCTAC  R1:CTCTTTGGTGAAGAGTTTTATGG  F2:CTGGCCACAGCACTTAAACA  R2:TCCGTGCGAGAATAATGATG |
| P16 | MELAS | MT-TL1 m.3243A>G del (35.65% ) | F:AATCCAGGTCGGTTTCTATCTAC  R:CTCTTTGGTGAAGAGTTTTATGG |
| P17 | MM | chrM: 315-16185 del (14.96%) | F:GTATGCACGCGATAGCATTG  R: GCACTCTTGTGCGGGATATT |
| P18 | MM | chrM:316-16185 del (18.25%) | F:GTATGCACGCGATAGCATTG  R: GCACTCTTGTGCGGGATATT |
| P19 | MM | - | - |
| P20 | IIM | chrM:3264-12299 del (12.60%) | F:GGGATAACAGCGCAATCCTA  R:TGGCTCAGTGTCAGTTCGAG |
| P21 | MM | - | - |
| P22 | MM | - | - |
| P23 | MM | - | - |
| P24 | MM | chrM:3264-16068 del (18.25%) | F:GGGATAACAGCGCAATCCTA  R:GCACTCTTGTGCGGGATATT |
|  |  | chrM:2167-13923 del (8.23%) | F:GTGGGAAGATTTATAGGTAGAGG  R:TGCGGGGGCTTTGTATGATTATG |
| P25 | MM | chrM:3264-14413 del (18.42%) | F:GGCATAACACAGCAAGACGA  R:CGTGAAGGTAGCGGATGATT |
| P26 | MM | - | - |
| P27 | MM | chrM:351-16071 del (24.03%) | F:GAGCTCTCCATGCATTTGGT  R:GTGGCTTTGGAGTTGCAGTT |
|  |  | chrM:3273-15882 del (12.81%) | F:TGCAGCCGCTATTAAAGGTT  R:GTGGCTTTGGAGTTGCAGTT |
|  |  | chrM:1693-13923 del (9.13%) | F:AAGGTGTAGCCCATGAGGTG  R:GAGAGGGGTCAGGGTTGATT |
|  |  | chrM:1773-14262 del (7.80%) | F:GGTCGAAGGTGGATTTAGCA  R:TTTAGGGGGAATGATGGTTG |
| P28 | MM | chrM:3264-13923 del (8.69%) | F:GGCATAACACAGCAAGACGA  R:AGGTAGGATTGGTGCTGTGG |
|  |  | chrM:3264-16072 del (27.77%) | F:GGCATAACACAGCAAGACGA  R:GGGAACGTGTGGGCTATTTA |

Abbreviations: C, control; P, patient; CPEO, chronic progressive external ophthalmoplegia; MELAS, mitochondrial encephalomyopathy, lactic acidosis and stroke-like episodes; MM, mitochondrial myopathy; IIM, idiopathic inflammatory myopathy.
