# Supplementary material for: HiFi long-read amplicon sequencing for full-spectrum variants of human mtDNA
Source: BMC Genomics. 2024 May 31;25:538. doi: 10.1186/s12864-024-10433-9 (PMC11141058; doi:10.1186/s12864-024-10433-9)
Supplement: Supplementary file 6 — Supplementary Material 6 [file 12864_2024_10433_MOESM6_ESM.docx]

**Table S2. Comparision of TGS and NGS in identifying heterogeneity loci.**

| NO | cHGVS | Zygosity | DP | VD | ARatio | FunctionName | Amino_acids | GeneSym | ExInID | Mitomap_Disease | HGMD  Pred |
| --- | --- | --- | --- | --- | --- | --- | --- | --- | --- | --- | --- |
| P1-TGS | m.5178C>A | Het | 7079 | 335 | 0.047 | missense | Leu/Met | MT-ND2 | STS | Longevity-/-Extraversion-/-diabetes-/-AMS-protection-/-blood-iron-metabolism-/-correlation-with-myocardial-infarction-/-atherosclerosis | Reported |
| P1-NGS | m.5178C>A | Het | 7923 | 368 | 0.046 | missense | Leu/Met | MT-ND2 | STS | Longevity-/-Extraversion-/-diabetes-/-AMS-protection-/-blood-iron-metabolism-/-correlation-with-myocardial-infarction-/-atherosclerosis | Reported |
| P1-TGS | m.8794C>T | Het | 7037 | 78 | 0.011 | missense | His/Tyr | MT-ATP6 | STS | Exercise-Endurance-/-Coronary-Atherosclerosis-risk | Reported |
| P1-NGS | m.8794C>T | Het | 7627 | 107 | 0.014 | missense | His/Tyr | MT-ATP6 | STS | Exercise-Endurance-/-Coronary-Atherosclerosis-risk | Reported |
| P1-TGS | m.12705C>T | Het | 7650 | 419 | 0.055 | coding-synon | Ile | MT-ND5 | CDS | Possible-protective-factor-for-normal-tension-glaucoma | Reported |
| P1-NGS | m.12705C>T | Het | 7016 | 432 | 0.062 | coding-synon | Ile | MT-ND5 | CDS | Possible-protective-factor-for-normal-tension-glaucoma | Reported |
| P1-TGS | m.14668C>T | Het | 7746 | 373 | 0.048 | coding-synon | Met | MT-ND6 | CDS | Depressive-Disorder-associated | Reported |
| P1-NGS | m.14668C>T | Het | 6599 | 304 | 0.046 | coding-synon | Met | MT-ND6 | CDS | Depressive-Disorder-associated | Reported |
| P3-TGS | m.195T>C | Het | 3692 | 53 | 0.014 | upstream | - | MT-ND2 | D-loop | BD-associated-/-melanoma-pts | Reported |
| P3-NGS | m.195T>C | Het | 5123 | 57 | 0.011 | upstream | - | MT-ND2 | D-loop | BD-associated-/-melanoma-pts | Reported |
| P3-TGS | m.10005A>G | Het | 1615 | 24 | 0.015 | upstream | - | MT-TG | tRNA | Hearing-loss-patient | Reported |
| P3-NGS | m.10005A>G | Het | 6640 | 77 | 0.012 | upstream | - | MT-TG | tRNA | Hearing-loss-patient | Reported |
| P3-TGS | m.11084A>G | Het | 1708 | 23 | 0.013 | missense | Thr/Ala | MT-ND4 | CDS | AD+-PD-MELAS | Conflicting-reports |
| P3-NGS | m.11084A>G | Het | 6447 | 72 | 0.011 | missense | Thr/Ala | MT-ND4 | CDS | AD+-PD-MELAS | Conflicting-reports |
| P10-TGS | m.150C>T | Het | 10332 | 235 | 0.023 | upstream | - | MT-ND2 | D-loop | Longevity-/-Cervical-Carcinoma-/-HPV-infection-risk | Conflicting-reports |
| P10-NGS | m.150C>T | Het | 14976 | 222 | 0.015 | upstream | - | MT-ND2 | D-loop | Longevity-/-Cervical-Carcinoma-/-HPV-infection-risk | Conflicting-reports |
| P10-TGS | m.16129G>A | Het | 9945 | 245 | 0.025 | upstream | - | MT-ND6 | D-loop | Cyclic-Vomiting-Syndrome-with-Migraine | Reported |
| P10-NGS | m.16129G>A | Het | 37434 | 532 | 0.014 | upstream | - | MT-ND6 | D-loop | Cyclic-Vomiting-Syndrome-with-Migraine | Reported |
| P10-TGS | m.16192C>T | Het | 10330 | 299 | 0.029 | upstream | - | MT-ND6 | D-loop | Melanoma-patients | Reported |
| P10-NGS | m.16192C>T | Het | 36170 | 536 | 0.015 | upstream | - | MT-ND6 | D-loop | Melanoma-patients | Reported |
| P12-TGS | m.3242G>A | Het | 11318 | 124 | 0.011 | upstream | - | MT-TL1 | tRNA | MM-/-HCM+renal-tubular-dysfunction | Reported |
| P12-NGS | m.3242G>A | Het | 14573 | 171 | 0.012 | upstream | - | MT-TL1 | tRNA | MM-/-HCM+renal-tubular-dysfunction | Reported |
| P12-TGS | m.6962G>A | Het | 2500 | 40 | 0.016 | coding-synon | Leu | MT-CO1 | CDS | Possible-helper-variant-for-15927A | Reported |
| P12-NGS | m.6962G>A | Het | 731 | 16 | 0.022 | coding-synon | Leu | MT-CO1 | CDS | Possible-helper-variant-for-15927A | Reported |
